# Exercise-based rehabilitation interventions for kinesiophobia after total knee arthroplasty: a systematic review and meta-analysis of randomized controlled trials

**DOI:** 10.1186/s12891-026-10640-3

**Published:** 2026-10-21

**Authors:** Hongbo Wen, Ting Zhang, Tingting Xie, Huiqin Xue

**Affiliations:** 1 Inner Mongolia Medical University, Inner Mongolia Autonomous Region, Hohhot, 010059 China; 2 The Second Affiliated Hospital of Inner Mongolia Medical University, Inner Mongolia Autonomous Region, Hohhot, 010010 China

**Keywords:** Total knee arthroplasty, Kinesiophobia, Exercise therapy, Physical therapy, Postoperative rehabilitation, Meta-analysis

## Abstract

**Background:**

Affecting 21–35% of patients after total knee arthroplasty (TKA), kinesiophobia critically impedes postoperative rehabilitation. Whether exercise-based interventions reduce kinesiophobia more effectively than routine postoperative rehabilitation remains unclear.

**Methods:**

We systematically searched 11 databases from inception to July 11, 2026, for RCTs comparing exercise-based rehabilitation with routine postoperative rehabilitation in adults undergoing primary TKA. The primary outcome was kinesiophobia. Secondary outcomes included knee function, pain, balance, mobilization time, and adherence. Random-effects meta-analyses were performed using RevMan 5.4. Certainty of evidence was assessed using GRADE.

**Results:**

Eleven RCTs (*N* = 1,096) were included. Exercise-based rehabilitation was associated with a reduction in kinesiophobia (SMD = − 1.22, 95% CI [− 1.72, − 0.72]; *I*^*2*^ = 93%; low certainty) and improvements in knee function (SMD = 1.19, 95% CI [0.67, 1.71]; *I*^*2*^ = 93%; low certainty). For pain, the ≤ 4-week pooled estimate (SMD = − 1.09, 95% CI [− 2.03, − 0.14]; *I*^*2*^ = 94%) was not interpretable due to extreme heterogeneity and was not robust: after exclusion of the single acute in-hospital trial, the confidence interval crossed zero (SMD = − 1.09, 95% CI [− 2.28, 0.09]). Leave-one-out sensitivity analyses otherwise supported the directional conclusions for kinesiophobia and knee function, although effect sizes were unstable and certainty remained low.

**Conclusion:**

Low-certainty evidence suggests that exercise-based rehabilitation may be associated with reductions in kinesiophobia at ≤ 4 weeks and an interpretable 12-week improvement in knee function after TKA; the ≤ 4-week and ≥ 24-week functional subgroup estimates were not interpretable because of extreme heterogeneity, and the early pain signal was possible but non-robust. Effect sizes should be interpreted with caution due to substantial heterogeneity, risk of bias, and sensitivity to outlying studies. Well-designed, multicenter RCTs with rigorous blinding and standardized protocols are urgently needed.

**Supplementary Information:**

The online version contains supplementary material available at https://doi.org/10.1186/s12891-026-10640-3.

## Introduction

Knee osteoarthritis, a prevalent degenerative joint disease, substantially compromises mobility and quality of life in older adults. Total knee arthroplasty (TKA) is the standard treatment for end-stage cases [1–3]. Postoperatively, between 21.75% and 35% of patients experience kinesiophobia [2, 4], which can significantly hinder their recovery [5, 6].

Kinesiophobia is an independent predictor of unfavorable outcomes following TKA [1, 7, 8] and may lead to cessation of rehabilitation exercises, postponement of early mobilization, and increased dependency in daily activities [5, 6]. Despite progressive refinements in perioperative care and rehabilitation protocols, targeted rehabilitation strategies for kinesiophobia, a complex psychological phenomenon, remain underdeveloped [9, 10]. Although standard postoperative rehabilitation includes physical therapy directed at restoring joint mobility and muscular strength, it typically fails to incorporate exercise-based rehabilitation protocols specifically designed to target and mitigate kinesiophobia, thereby leaving the psychological barriers to movement largely unaddressed [11–14].

Emerging evidence underscores the significant impact of psychological factors, including kinesiophobia, on functional recovery after TKA [3, 8, 15]. While traditional physical therapy enhances physical recovery, it often fails to adequately address psychological barriers [16]. In contrast, exercise-based rehabilitation interventions, which integrate targeted physical conditioning with psychological techniques, have demonstrated promising benefits in reducing kinesiophobia and promoting recovery in patients with knee osteoarthritis [12, 13, 17, 18]. Such interventions frequently incorporate progressive muscle relaxation, motor imagery, elastic band resistance training, and virtual reality-based relaxation exercises: modalities specifically aimed at addressing psychological contributors to postoperative outcomes [19, 20]. However, despite these encouraging results, few studies have directly compared the effectiveness of exercise-based interventions with traditional physical therapy in reducing postoperative kinesiophobia after TKA, and there is a lack of comprehensive, systematic integrative analysis.

This gap underscores the need for a more rigorous investigation. Accordingly, we conducted a systematic review and meta-analysis to evaluate the comparative effectiveness of exercise-based rehabilitation versus routine postoperative rehabilitation in reducing postoperative kinesiophobia among patients with knee osteoarthritis undergoing TKA. The findings aim to inform and refine future postoperative care strategies.

## Methods

### Study design

This systematic review and meta-analysis was reported in accordance with the PRISMA 2020 guidelines [21] and registered with PROSPERO (registration number CRD420261461385).

### Search strategies

We systematically searched PubMed, Embase, Cochrane Library, Web of Science, Ovid MEDLINE, Scopus, CINAHL, CNKI, VIP, Wanfang, and CBM for RCTs comparing exercise-based rehabilitation with control interventions in adults undergoing primary TKA. Control interventions consisted of routine postoperative rehabilitation. Reference lists of relevant articles were manually searched. The search period spanned from inception to July 11, 2026. Search terms combined MeSH headings and free-text terms for TKA (“Arthroplasty, Replacement, Knee“[Mesh] OR “total knee arthroplasty” OR “TKA”) AND exercise interventions (“Exercise“[Mesh] OR “Exercise Therapy“[Mesh] OR “Physical Therapy Modalities“[Mesh] OR “Relaxation Therapy“[Mesh] OR exercise* OR “motor imagery” OR “progressive muscle relaxation” OR “resistance training” OR “elastic band exercise” OR “virtual reality exercise”). See Supplementary Table S1 for detailed search strategies. No language restriction was applied during the database search; however, studies were eligible for inclusion only if published in Chinese or English.

### Inclusion and exclusion criteria

Articles were included if they: (1) enrolled adults (≥ 18 years) undergoing primary TKA; (2) were RCTs comparing exercise-based rehabilitation (including progressive muscle relaxation, motor imagery, resistance training, virtual reality-based exercise, or self-management exercise education with structured physical exercise) versus routine postoperative rehabilitation or usual care; interventions were considered “exercise-based” if they included a planned, progressive, and monitored physical activity component, regardless of whether they were delivered in a supervised facility-based or home-based setting; (3) were published in Chinese or English; and (4) reported Tampa Scale of Kinesiophobia (TSK) as the primary outcome. Secondary outcomes were not required for study inclusion but were extracted when reported.

Exclusion criteria: (1) arthroscopy, ACL reconstruction, or revision knee surgery; (2) observational, retrospective, or non-randomized studies; (3) duplicate publications or insufficient data; (4) conference abstracts, dissertations, or grey literature.

### Outcomes

Primary outcome: Kinesiophobia was measured using the TSK, including the TSK-11, TSK-13, and TSK-17 versions. The TSK is a validated self-report instrument assessing fear of movement or reinjury, with higher scores indicating greater kinesiophobia. Psychometric studies support its reliability and validity in musculoskeletal and post-surgical populations, although the versions differ in item content and factor structure.

Secondary outcomes: Knee joint function was assessed using the Western Ontario and McMaster Universities Osteoarthritis Index (WOMAC), Knee Society Score (KSS), Hospital for Special Surgery (HSS) score, or Knee Injury and Osteoarthritis Outcome Score (KOOS). Pain was measured using the Visual Analog Scale (VAS) or Numerical Rating Scale (NRS). Balance was measured using the Berg Balance Scale (BBS). Time to first mobilization after surgery was recorded in hours. Rehabilitation adherence was recorded as reported by study authors.

### Literature screening

After removing duplicate literatures using EndNote 21.3 software, two researchers (H.W., T.Z.) independently read the titles and abstracts of the literatures and conducted preliminary screening in accordance with the inclusion and exclusion criteria to eliminate literatures that obviously failed to meet the criteria. Subsequently, full-text reading was performed on literatures passing the preliminary screening for secondary screening to identify the finally included studies. Disagreements arising during the screening process were resolved through negotiation; if consensus could not be reached, a third researcher (H.X.) was consulted for joint discussion to decide on inclusion.

### Data extraction

Data were independently extracted by two researchers and cross-checked. The extracted information included the first author, year of publication, country, study design, sample size, age and gender of research subjects, follow-up duration, specific details of intervention and control measures, outcome indicators, and information related to risk of bias assessment. Clinical outcomes were summarized in an Excel spreadsheet for further analysis to compare the intervention effects across different studies. If a study did not directly report the mean and standard deviation, the method described by Wan et al. [22] was adopted to estimate these values based on the median, interquartile range, or extreme values. Among the 11 included studies, only one study [17] reported TSK scores as median (IQR) for the primary outcome and therefore required Wan et al. [22] imputation; the remaining 10 studies reported TSK scores directly as mean ± SD.

### Quality assessment

Quality assessment was conducted using the revised Cochrane risk-of-bias tool for randomized trials (RoB 2) [23], evaluating five domains: randomization process, deviations from intended interventions, missing outcome data, measurement of the outcome, and selection of the reported result. Each domain was rated as low risk, some concerns, or high risk. Two researchers independently assessed each study; disagreements were resolved with a third researcher.

### Methodological quality assessment

The certainty of evidence for each outcome was assessed using the GRADE approach [24]. Two researchers independently rated certainty for each outcome across five domains: risk of bias, inconsistency, indirectness, imprecision, and publication bias. Each domain was rated as having no serious concerns, serious concerns, or very serious concerns, and overall certainty was downgraded by one or two levels accordingly. Evidence was classified as High, Moderate, Low, or Very Low certainty. Ratings were based on the meta-analyses conducted for each outcome. Disagreements were resolved by consensus with a third researcher.

### Data analysis

Meta-analyses were performed using RevMan 5.4 software provided by the Cochrane Collaboration. Continuous data were expressed as mean difference (MD) or standardized mean difference (SMD) with their 95% confidence intervals (CIs), while dichotomous data were presented as odds ratio (OR) and 95% CIs. A P value less than 0.05 was considered statistically significant. Statistical heterogeneity across studies was assessed using the Chi^2^ test and *I*^*2*^ statistics. A random-effects model was used as the primary analysis to account for anticipated clinical diversity; a fixed-effect model was applied as a sensitivity analysis when heterogeneity was low (*P* > 0.10 and *I*^*2*^ < 50%). Subgroup analyses were conducted to explore sources of heterogeneity, and sensitivity analyses were carried out by omitting individual studies one by one to verify the stability of pooled results. Outcomes reported by only one study were descriptively analyzed. SMD with 95% CIs was employed for kinesiophobia, knee function, and pain to harmonize the different scale versions used across studies. To ensure consistent directionality across all functional outcomes, WOMAC scores were inverted (multiplied by − 1) prior to pooling, so that positive effect estimates uniformly favored the exercise intervention group. This alignment was necessary because WOMAC scores decrease with improved function, whereas HSS, KOOS and KSS scores increase with improved function. We had planned to explore sources of substantial heterogeneity (*I*^*2*^ > 75%) using meta-regression with covariates including mean age, proportion of female participants, total intervention duration in weeks, and total number of exercise sessions. However, this analysis could not be undertaken because the limited number of included studies (< 10 for most subgroups) would have resulted in insufficient statistical power. Subgroups with extreme heterogeneity (*I*^*2*^ > 90%) were considered statistically not interpretable. This rule applies to subgroup and sensitivity analyses; overall pooled estimates are reported with explicit caution and GRADE downgrading. For these subgroups, only the number of studies, sample size, and *I*^*2*^ are reported in the main text; pooled effect estimates and 95% CIs are presented in Supplementary Table S3 for transparency only and are not interpreted as significance-tested findings.

## Results

### Search results

**Fig. 1:**
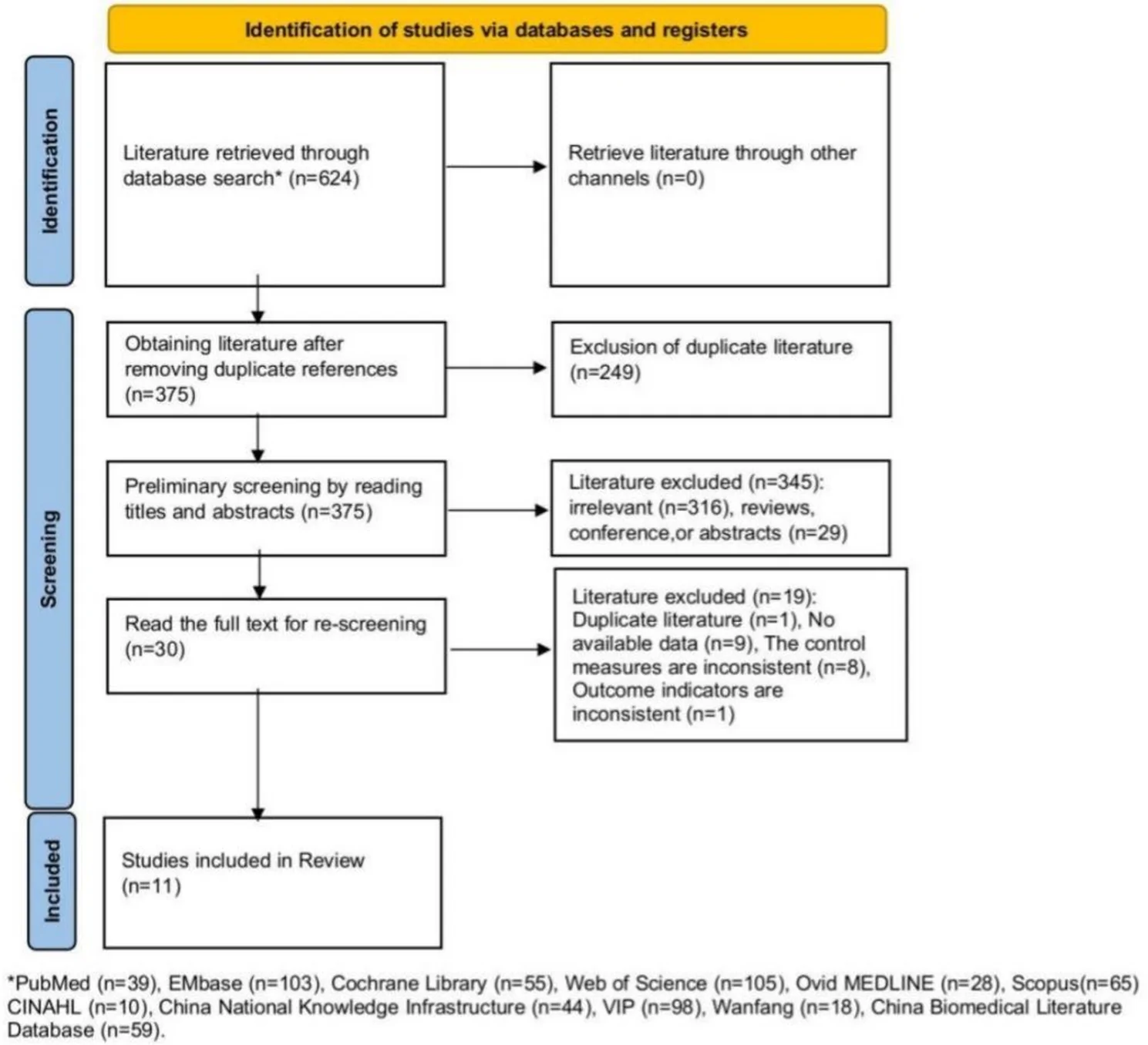
PRISMA flowchart

An initial search yielded 624 articles. Using EndNote 21.3 software, 249 duplicate articles were excluded. After reviewing the titles and abstracts, 316 articles that did not meet the criteria and 29 review articles were excluded, leaving 30 articles for full‑text screening. Nineteen full-text articles were excluded (duplicate: *n* = 1; insufficient data: *n* = 9; ineligible control: *n* = 8; inconsistent outcomes: *n* = 1). The complete list of excluded studies with reasons is provided in Supplementary Table S2, resulting in the inclusion of 11 RCTs [13, 17, 19, 25–32]. Two researchers independently screened all full texts. Disagreements during full-text selection were resolved by consensus or by consulting a third reviewer. The screening process is shown in Fig. 1.

### Basic information of the included literature

Eleven RCTs (*N* = 1,096: 550 intervention, 546 control) [13, 17, 19, 25–32] were included. Study characteristics are summarized in Table 1.

**Table 1:** Basic information of the included studies

Study	Country	Sample Size(T / C)	Sample Age (Mean ± SD, T / C)	Sex (M / F)	Intervention	Control	Follow-up (weeks)	Outcome Measures
Monticone 2013^*^[25]	Italy	55 / 55	67.0 ± 6.1 / 68.0 ± 7.1	40 / 70	Prescribed progressive home-based exercise program (quadriceps strengthening, ROM exercises, functional task training) + educational handbook for kinesiophobia management + monthly telephone follow-ups for adherence reinforcement	Routine postoperative rehabilitation	48	TSK−13, KOOS, NRS
Li 2020 [26]	China	48 / 47	65.04 ± 7.16 / 63.87 ± 10.58	19 / 76	Routine postoperative rehabilitation + Otago exercise training	Routine postoperative rehabilitation	12	TSK−17, BBS, HSS
Zhang 2020 [27]	China	45 / 45	76.3 ± 3.9 / 77.8 ± 4.2	43 / 47	Fear-avoidance model-guided early staged functional training	Routine postoperative rehabilitation	24	TSK−17, KSS
Li 2022 [28]	China	49 / 49	65.18 ± 3.19 / 63.34 ± 3.17	51 / 47	Routine postoperative rehabilitation + preoperative & early postoperative standardized quantitative rehabilitation training + whole-course standardized pain management	Routine postoperative rehabilitation	24	TSK−17, Time to first mobilization, Adherence
Çetinkaya 2022 [13]	Turkey	30 / 30	NR	7 / 53	Routine postoperative rehabilitation + resistance band training	Routine postoperative rehabilitation	4	TSK−17, VAS
Eymir 2022 [17]	Turkey	55 / 51	67.17 ± 9.53 / 65.67 ± 10.69	26 / 80	Routine postoperative rehabilitation + progressive muscle relaxation (PMR) training	Routine postoperative rehabilitation	12	TSK−17, HSS, NPRS
Bai 2024 [29]	China	100 / 100	63.12 ± 5.28 / 62.89 ± 5.18	85 / 115	Routine postoperative rehabilitation + whole-course Swiss ball dynamic core training	Routine postoperative rehabilitation	12	TSK−11, VAS, HSS, BBS, Adherence
Wang 2025 [31]	China	54 / 53	62.8 ± 5.4 / 62.5 ± 5.7	59 / 48	Routine postoperative rehabilitation + mirror neuron theory-based rehabilitation training	Routine postoperative rehabilitation	24	TSK−17, NRS, KSS, HSS, Adherence, Time to first mobilization
Yin 2025 [32]	China	41 / 41	66.00 ± 7.59 / 67.34 ± 8.19	34 / 48	Routine postoperative rehabilitation + mixed reality (MR)-guided exercise training	Routine postoperative rehabilitation	12	TSK−17, HSS
Kara 2026 [19]	Turkey	53 / 55	67.0 ± 5.5 / 66.0 ± 8.7	12 / 96	Routine postoperative rehabilitation + mobile App education & exercise programs (My Knee Prosthesis and My Health)	Routine postoperative rehabilitation	4	TSK−17, VAS
Núñez-Cortés 2024 [30]	Spain	20 / 20	70.6 ± 6.9 / 71.6 ± 7.2	23 / 17	Routine postoperative rehabilitation + high-intensity resistance band training	Routine postoperative rehabilitation	0.4	TSK−11, VAS, WOMAC

T: intervention group (exercise-based rehabilitation); C: control group (routine postoperative rehabilitation); SD: standard deviation; M: male; F: female; NR: not reported; TSK: Tampa Scale for Kinesiophobia (versions TSK‑11, TSK‑13, or TSK‑17 as indicated); KOOS: Knee Injury and Osteoarthritis Outcome Score; NRS: Numerical Rating Scale; BBS: Berg Balance Scale; HSS: Hospital for Special Surgery score; KSS: Knee Society Score; VAS: Visual Analog Scale; NPRS: Numerical Pain Rating Scale; FPS‑R: Faces Pain Scale‑Revised; WOMAC: Western Ontario and McMaster Universities Osteoarthritis Index; PMR: progressive muscle relaxation; MR: mixed reality. Follow-up duration was defined as the time from surgery to the final outcome assessment. For Chinese-language studies, the original intervention names are retained in the table; detailed descriptions are provided in the main text. Missing values (NR) indicate that the original publication did not report the corresponding data

^*^ Although Monticone et al. included educational components, this study was classified as exercise-based rehabilitation because the intervention comprised a structured, progressive home exercise program (quadriceps strengthening, range-of-motion, functional training) with defined progression criteria, individually prescribed and objectively monitored via telephone follow-ups

### Quality evaluation results

**Fig. 2:**
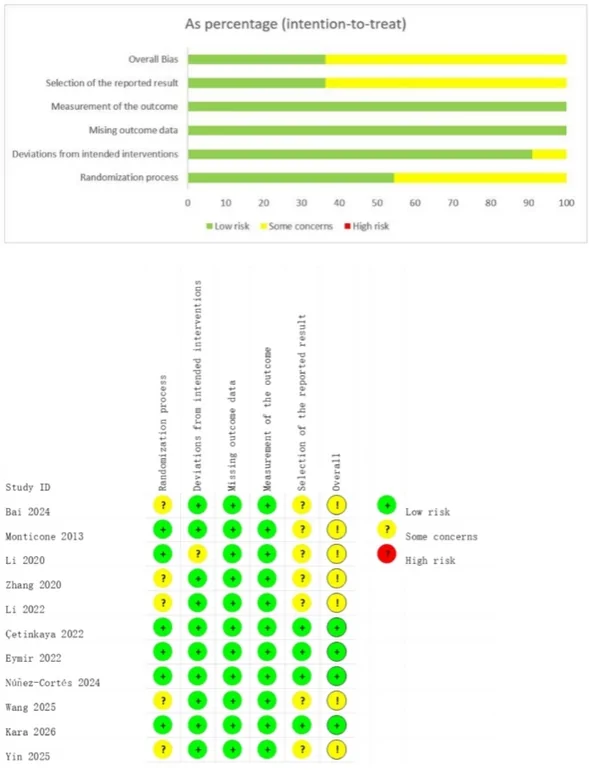
Risk of bias summary (RoB 2). ‘+’ low risk; ‘?’ some concerns; ‘−’ high risk

Risk of bias was assessed using RoB 2 across five domains. Four of the 11 included studies (36%) [13, 17, 19, 30] were judged as low overall risk of bias, seven studies (64%) [25–29, 31, 32] raised some concerns. No studies were rated as high overall risk. Common concerns included absence of allocation concealment (5 studies) [27–29, 31, 32] and lack of pre-specified analysis plans (7 studies) [25–29, 31, 32]. All studies were rated low risk for missing outcome data and outcome measurement. Due to the nature of rehabilitation interventions, no study blinded participants or personnel. This represents an important source of performance bias that may have inflated effect sizes for subjective outcomes, as reflected in the GRADE certainty downgrade (Fig. 2).

### Meta-analysis results

#### Kinesiophobia

**Fig. 3:**
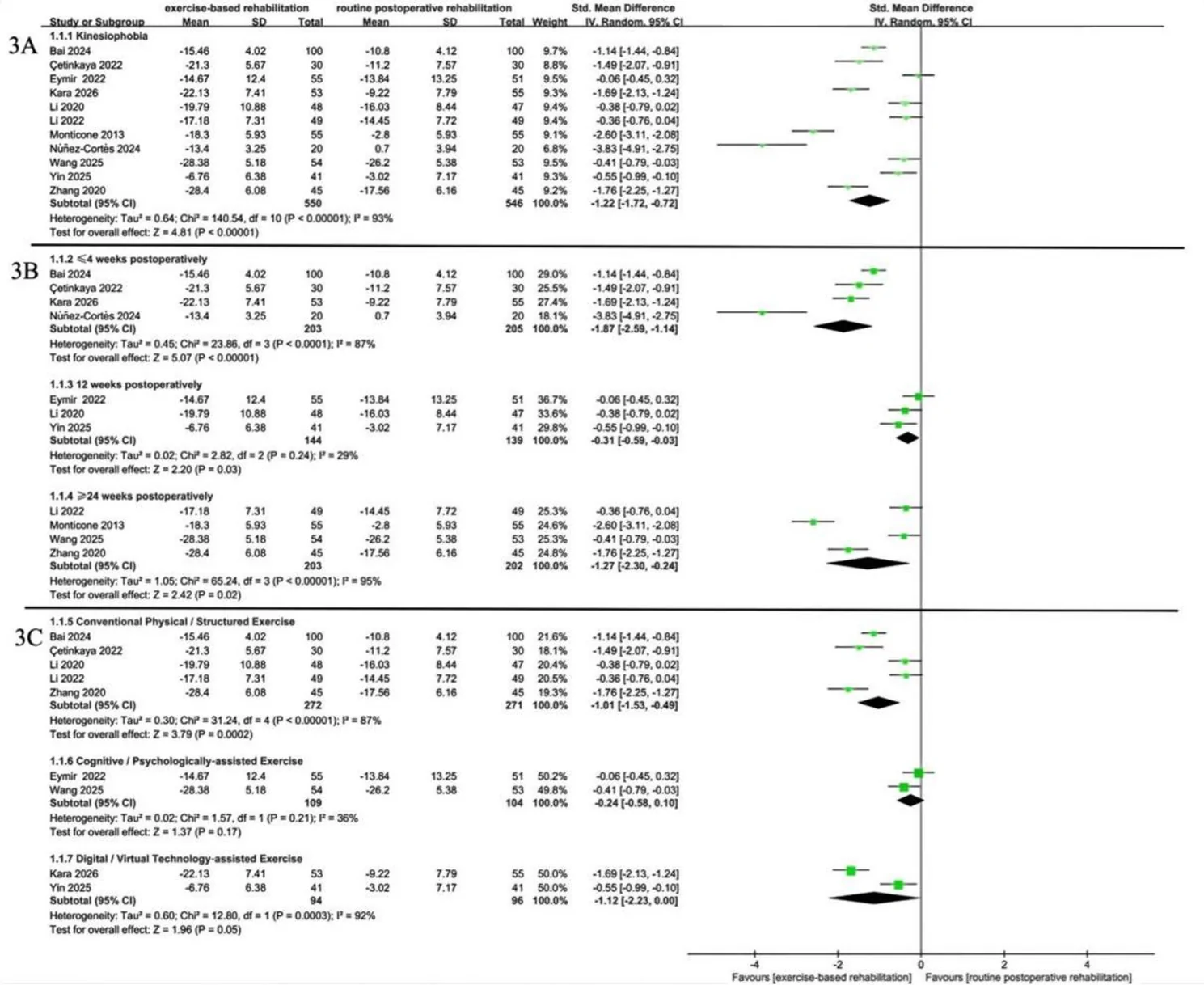
Forest plot of the effect of exercise-based rehabilitation on kinesiophobia after TKA. (3 A) Overall analysis; (3B) Subgroup analysis by follow-up time; (3 C) Subgroup analysis by intervention type. Subgroups with *I*^*2*^ > 90% are not interpretable; see Supplementary Table S3

Eleven studies (*n* = 1,096) [13, 17, 19, 25–32] assessed kinesiophobia. Of these, 4 had low overall risk of bias [13, 17, 19, 30] and 7 had some concerns [25–29, 31, 32]. Using a random-effects model (*I*^*2*^ = 93%), exercise-based rehabilitation was associated with a reduction in kinesiophobia compared with routine postoperative rehabilitation (SMD = − 1.22, 95% CI [− 1.72, − 0.72]; Fig. 3A).

##### Subgroup analyses

By follow-up time (Fig. 3B), the effect was most pronounced at ≤ 4 weeks (k = 4, *n* = 408, SMD = − 1.87, 95% CI [− 2.59, − 1.14], *P* < 0.001, *I*^*2*^ = 87%) and attenuated at 12 weeks (k = 3, *n* = 283, SMD = − 0.31, 95% CI [− 0.59, − 0.03], *P* = 0.03, *I*^*2*^ = 29%). At ≥ 24 weeks, four trials (*n* = 405) contributed data; *I*^*2*^ = 95%, not interpretable (Supplementary Table S3). By intervention modality (Fig. 3C), conventional exercise (k = 5, *n* = 543, SMD = − 1.01, 95% CI [− 1.53, − 0.49], *P* = 0.0002, *I*^*2*^ = 87%). For digital/technology-assisted exercise, two trials (*n* = 190) contributed data; *I*^*2*^ = 92%, not interpretable (Supplementary Table S3). Cognitive/psychologically-assisted exercise showed no significant reduction (k = 2, *n* = 213, SMD = − 0.24, 95% CI [− 0.58, 0.10], *P* = 0.17, *I*^*2*^ = 36%)

#### Knee joint function

**Fig. 4:**
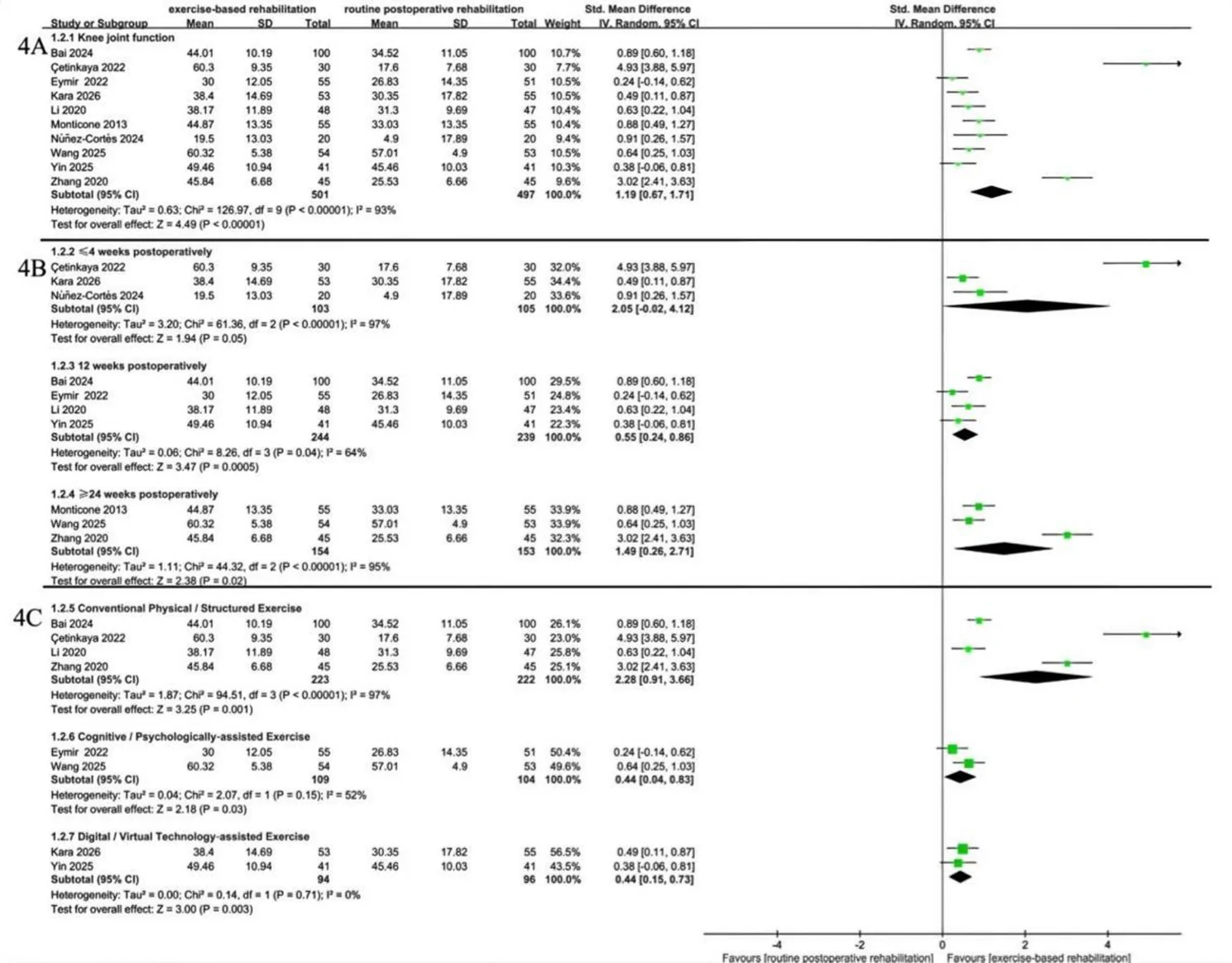
Forest plot of the effect of exercise-based rehabilitation on knee joint function after TKA. (4 A) Overall analysis; (4B) Subgroup analysis by follow-up time; (4 C) Subgroup analysis by intervention type. WOMAC scores were inverted prior to pooling such that positive SMD values uniformly indicate better knee function in the exercise-based rehabilitation group. Subgroups with *I*^*2*^ > 90% are not interpretable; see Supplementary Table S3

Ten studies (*n* = 998) [13, 17, 19, 25–27, 29–32] evaluated knee function. Of these, 4 had low overall risk [13, 17, 19, 30] and 6 had some concerns [25–27, 29, 31, 32]. Using a random-effects model (*I*^*2*^ = 93%), exercise-based rehabilitation was associated with improved knee function (SMD = 1.19, 95% CI [0.67, 1.71]; Fig. 4A).

##### Subgroup analyses

By follow-up time (Fig. 4B), three trials (*n* = 208) contributed data; *I*^*2*^ = 97%, not interpretable (Supplementary Table S3). Significant improvements emerged at 12 weeks (k = 4, *n* = 483, SMD = 0.55, 95% CI [0.24, 0.86], *P* = 0.0005, *I*^*2*^ = 64%). At ≥ 24 weeks, three trials (*n* = 307) contributed data; *I*^*2*^ = 95%, not interpretable (Supplementary Table S3). By modality (Fig. 4C), cognitive/psychologically-assisted exercise (k = 2, *n* = 213, SMD = 0.44, 95% CI [0.04, 0.83], *P* = 0.03, *I*^*2*^ = 52%) and digital/technology-assisted exercise (k = 2, *n* = 190, SMD = 0.44, 95% CI [0.15, 0.73], *P* = 0.003, *I*^*2*^ = 0%) were associated with improved function. For conventional exercise, four trials (*n* = 445) contributed data; *I*^*2*^ = 97%, not interpretable (Supplementary Table S3).

#### Pain

**Fig. 5:**
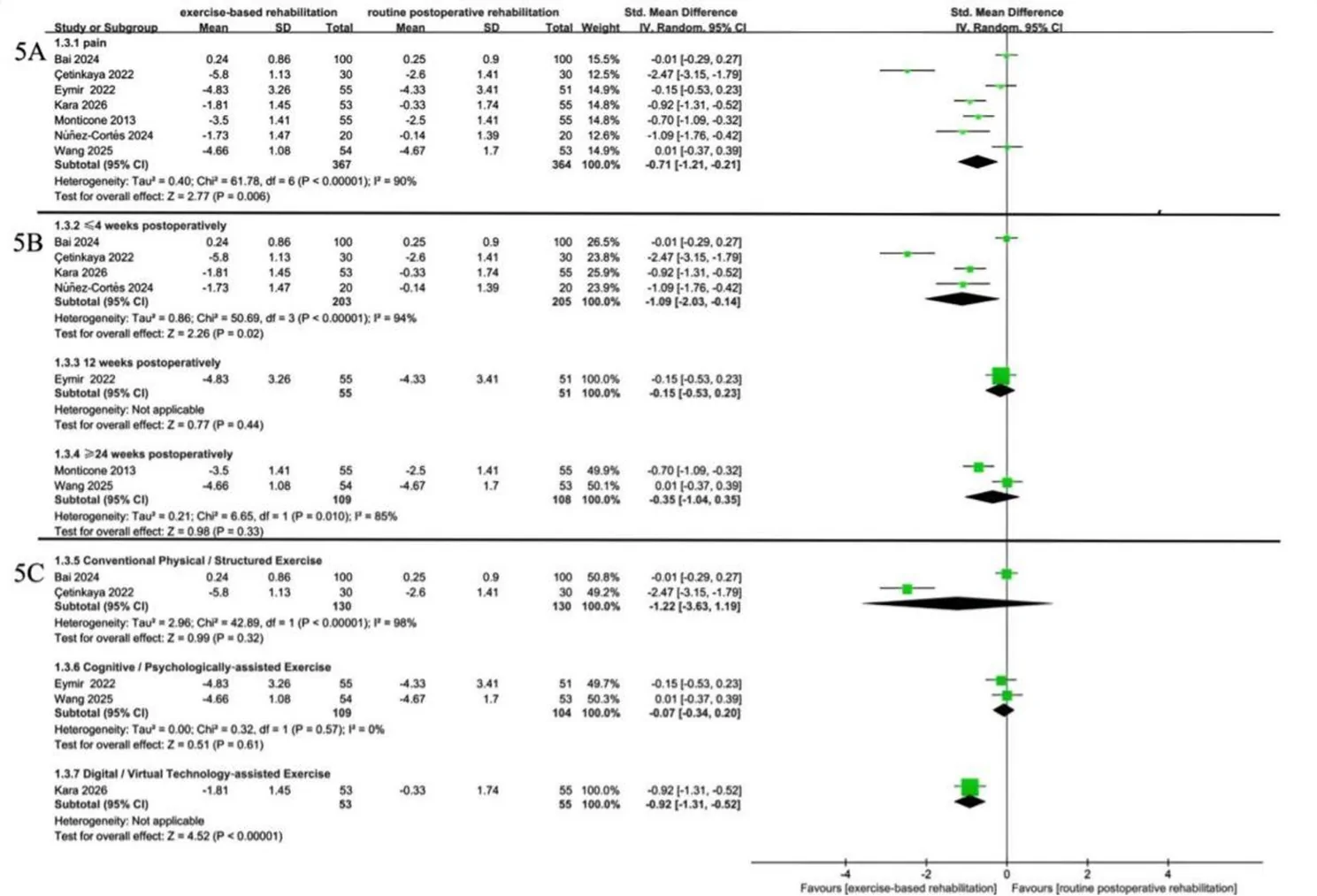
Forest plot of the effect of exercise-based rehabilitation on pain after TKA. (5 A) Overall analysis; (5B) Subgroup analysis by follow-up time; (5 C) Subgroup analysis by intervention type. Subgroups with *I*^*2*^ > 90% are not interpretable; see Supplementary Table S3

Seven studies (*n* = 731) [13, 17, 19, 25, 29–31] evaluated pain. Of these, 4 had low overall risk [13, 17, 19, 30] and 3 had some concerns [25, 29, 31]. Using a random-effects model (*I*^*2*^ = 90%), the pooled analysis suggested a possible reduction in pain favoring the exercise-based rehabilitation group (SMD = − 0.71, 95% CI [− 1.21, − 0.21]; Fig. 5A).

##### Subgroup analyses

At ≤ 4 weeks, four trials (*n* = 408) contributed data; *I*^*2*^ = 94%, not interpretable (Supplementary Table S3). This early effect was also not robust: after excluding Núñez-Cortés 2024 [30], the pooled estimate remained numerically similar (SMD = − 1.09) but the confidence interval crossed zero (95% CI [− 2.28, 0.09]); this estimate was also not interpretable due to extreme heterogeneity (*I*^*2*^ = 96%). At 12 weeks, only one study reported pain outcomes; Eymir et al. [17] found that the intervention group had a 0.5 point greater reduction in NPRS scores compared with the control group. At ≥ 24 weeks (k = 2, *n* = 217, SMD = − 0.35, 95% CI [− 1.04, 0.35], *P* = 0.33, *I*^*2*^ = 85%), the effect was non-significant. By modality (Fig. 5C), for conventional exercise, two trials (*n* = 260) contributed data; *I*^*2*^ = 98%, not interpretable (Supplementary Table S3). For cognitive/psychologically-assisted exercise, two trials (*n* = 213) contributed data; the pooled estimate was SMD = − 0.07, 95% CI [− 0.35, 0.21], *P* = 0.61, *I*^*2*^ = 0%. Digital/technology-assisted exercise was evaluated in a single study; Kara et al. [19] reported that the intervention group had a 1.48 point greater reduction in VAS scores compared with the control group.

#### Berg Balance Scale

**Fig. 6:**
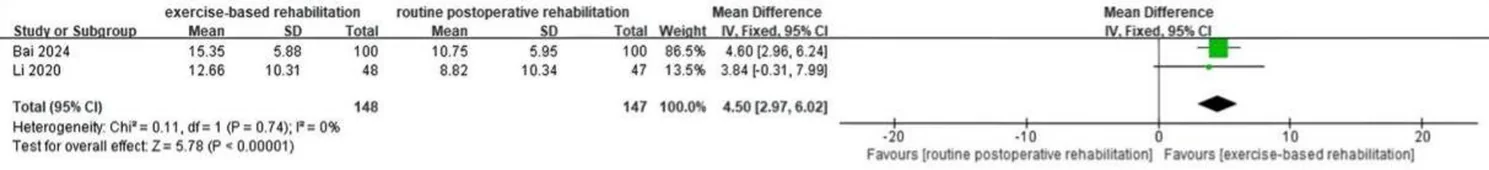
Forest plot of the effect of exercise-based rehabilitation on balance

Two studies [26, 29] (*n* = 295) evaluated balance using the Berg Balance Scale (BBS). Statistical heterogeneity was minimal (*I*^*2*^ = 0%, *P* = 0.74); therefore, a fixed-effect model was employed. The pooled results showed that exercise-based rehabilitation was associated with improved balance (MD = 4.50, 95% CI [2.97, 6.02], *P* < 0.001; Fig. 6). However, this estimate is based on only two small trials and was rated Very Low certainty by GRADE; the result should be interpreted as preliminary.

#### Time to first mobilization

**Fig. 7:**
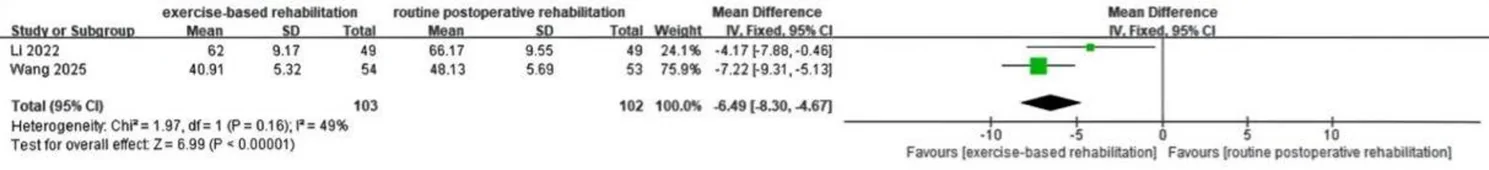
Forest plot of the effect of exercise-based rehabilitation on time to first mobilization

Two studies [28, 31] (*n* = 205) evaluated time to first mobilization after surgery. Moderate heterogeneity was observed across studies (*I*^*2*^ = 49%, *P* = 0.16); therefore, a fixed-effect model was employed. The pooled results showed that exercise-based rehabilitation was associated with shorter time to first mobilization (MD = − 6.49 h, 95% CI [− 8.30, − 4.67], *P* < 0.001; Fig. 7). This estimate was rated Very Low certainty by GRADE due to small sample size and risk of bias.

#### Rehabilitation adherence

**Fig. 8:**
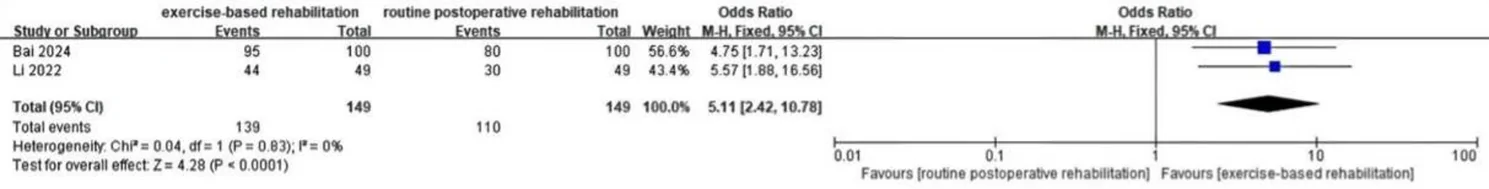
Forest plot of the effect of exercise-based rehabilitation on rehabilitation adherence

Two studies [28, 29] (*n* = 298) evaluated rehabilitation adherence. Statistical heterogeneity was minimal (*I*^*2*^ = 0%, *P* = 0.83); therefore, a fixed-effect model was employed. The pooled results showed that exercise-based rehabilitation was associated with higher rehabilitation adherence (OR = 5.11, 95% CI [2.42, 10.78], *P* < 0.001; Fig. 8). This estimate was rated Very Low certainty by GRADE and should be interpreted cautiously.

#### Sensitivity analysis

Leave-one-out sensitivity analyses were performed by sequentially omitting each study. For kinesiophobia, the direction of the pooled effect remained unchanged across all iterations (pooled SMD range: −1.34 to − 1.03, all *P* < 0.001). For knee function, the pooled effect also remained consistently significant (SMD range: 0.86 to 1.31). For pain, the overall pooled effect remained directionally stable across all leave-one-out iterations (SMD range: −0.84 to − 0.44), although all estimates were accompanied by high heterogeneity. These results indicate that no single study drove the direction of the overall conclusions for any of the primary outcomes.

Exclusion of Núñez-Cortés 2024 [30] (acute in-hospital trial) did not materially change the overall conclusions for kinesiophobia or knee function; the pooled SMDs remained directionally consistent and statistically significant (kinesiophobia: SMD = − 1.03, 95% CI [− 1.50, − 0.56], *I*^*2*^ = 92%; knee function: SMD = 1.22, 95% CI [0.66, 1.78], *I*^*2*^ = 94%). In the ≤ 4-week subgroup, however, the early pain reduction became non-significant after excluding Núñez-Cortés 2024 [30] (SMD = − 1.09, 95% CI [− 2.28, 0.09], *I*^*2*^ = 96%), with the point estimate nearly unchanged due to weighting balance, suggesting that early analgesic effects were partially driven by very-short-term inpatient training. Excluding Monticone 2013 [25] (a composite educational-exercise intervention) also did not materially alter the directional conclusions for kinesiophobia and knee function. However, all these sensitivity estimates were accompanied by extreme heterogeneity (*I*^*2*^ > 90%) and are not interpretable; numerical values are presented in Supplementary Table S3 for transparency only.

No single study altered the direction or significance of the overall kinesiophobia or knee-function estimates. For pain, the ≤ 4-week subgroup became non-significant after excluding Núñez-Cortés 2024 [30]; the early pain finding is therefore not robust. Given the low GRADE certainty and high heterogeneity, the pooled estimates should be interpreted cautiously.

#### Publication bias test

**Fig. 9:**
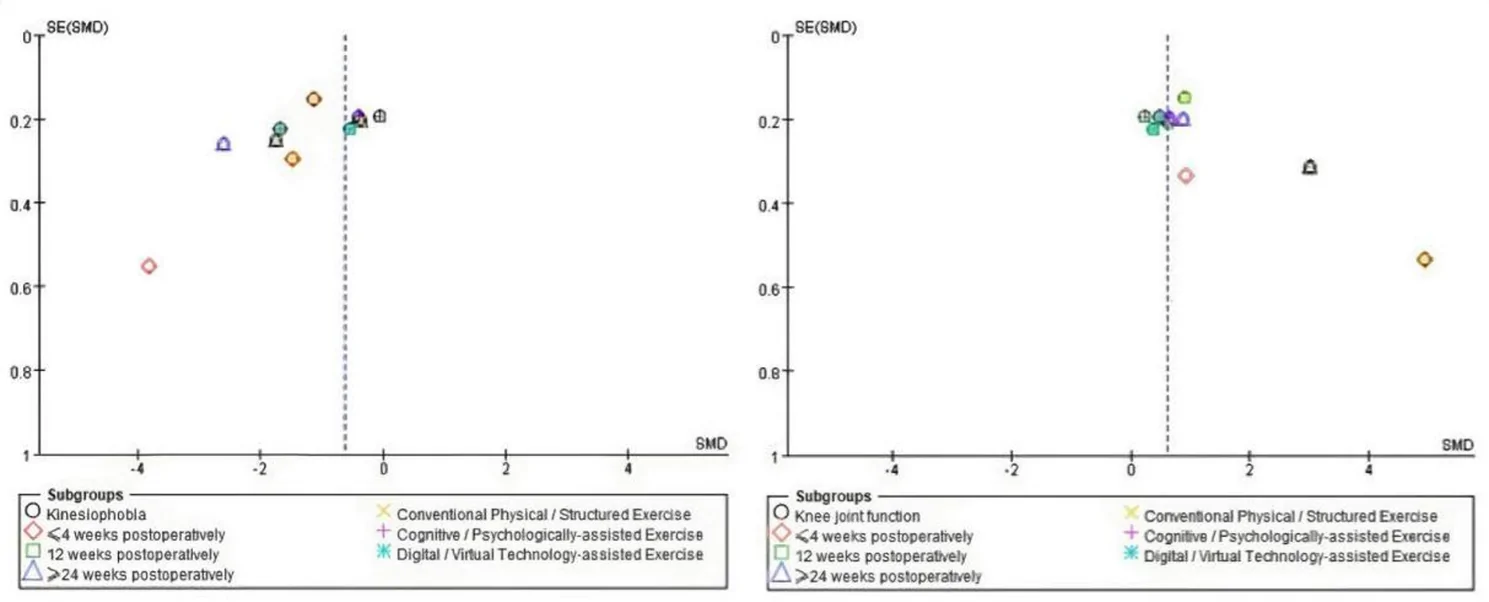
Funnel plot for the assessment of publication bias in kinesiophobia and knee joint function outcomes

Funnel plots for kinesiophobia and knee joint function appeared broadly symmetric(Fig. 9). However, with only 11 and 10 studies included for these respective outcomes, the statistical power of funnel plot asymmetry tests was low. We therefore cannot exclude the possibility of small-study effects or selective reporting, and the absence of apparent asymmetry should not be interpreted as evidence of no publication bias.

#### GRADE assessment

GRADE assessment rated the certainty of evidence as Low for all three critical outcomes (kinesiophobia, knee function, pain), downgraded by two levels for serious risk of bias and serious inconsistency (*I*^*2*^ ≥ 90%). For the three important outcomes (Berg Balance Scale, time to first mobilization, rehabilitation adherence), certainty was rated Very Low, driven by serious risk of bias and very serious imprecision from only two small trials each. Full domain-by-domain evidence profiles and downgrade rationales are provided in Supplementary Table S4.

Exercise-based rehabilitation may be associated with reductions in kinesiophobia at ≤ 4 weeks and an interpretable 12-week improvement in knee function; the ≤ 4-week and ≥ 24-week functional estimates were not interpretable, and the early pain reduction was a possible but non-robust signal. Clinical decision-makers should exercise caution when interpreting these results due to methodological flaws in the current evidence base and high heterogeneity or small sample sizes for some outcomes.

## Discussion

### Principal findings

This systematic review and meta-analysis synthesized data from 11 RCTs to evaluate the effects of exercise-based rehabilitation on postoperative recovery after TKA. While the pooled estimates suggested a possible association between exercise-based rehabilitation and improvements in kinesiophobia and knee function, the evidence is of low certainty. For knee function, only the 12-week subgroup estimate was interpretable; the ≤ 4-week and ≥ 24-week subgroup estimates were not interpretable because of extreme heterogeneity. The effect sizes are likely inflated due to lack of participant and personnel blinding, and the true effects, if they exist, are probably substantially smaller. These findings should be interpreted as hypothesis-generating only and cannot support definitive clinical recommendations. The apparent early benefits (≤ 4 weeks) are particularly uncertain given the high heterogeneity within the early functional and pain subgroups.

### Effects on kinesiophobia

Pre-specified subgroup analyses by postoperative time point revealed a clinically instructive temporal pattern: the effect was most pronounced at ≤ 4 weeks postoperatively and attenuated substantially at 12 weeks. At ≥ 24 weeks postoperatively, four trials contributed to this subgroup; however, *I*^*2*^ = 95% rendered the estimate not interpretable, and we therefore do not report a significance-tested long-term effect. Long-term conclusions remain tentative.

The acute phase (≤ 4 weeks) may represent a critical window during which pain catastrophizing beliefs are particularly malleable. During this period, graded motor exposure, by providing direct experiential evidence that movement is safe, may rapidly disrupt the fear-avoidance cycle. This is corroborated by Aleksić et al. [6], who found that 27% of TKA patients exhibited kinesiophobia on postoperative day 5, with multidimensional pain composite scores emerging as the strongest predictors. The mid-term attenuation (12 weeks) does not necessarily indicate that exercise loses efficacy; rather, it may reflect a ceiling effect, wherein control group patients experience natural habituation to movement through routine daily activities, thereby narrowing the intergroup gap. Longitudinal data from Deniz et al. [33] support this interpretation, showing that kinesiophobia increased at 3 months postoperatively compared to preoperative levels but decreased effectively by 12 months. Notably, the large effect observed at ≤ 4 weeks, while based on more studies than the long-term estimate, was also plagued by high heterogeneity and should not be interpreted as a robust, reproducible early treatment effect. It may reflect a combination of true short-term benefit, placebo effects due to unblinding, and baseline imbalance across small trials.

Regarding the pervasive heterogeneity, we attribute it largely to the marked variability in intervention dosage, including session duration, weekly frequency, and total program length, which ranged from 2.8 days of inpatient training [30] to 48 weeks of home-based regimens [25]. Sensitivity analyses confirmed that no single study dominated the direction of the pooled effect, and the overall conclusion remained robust across all leave-one-out iterations. This distinction is important: the directional conclusion is robust, but the precise magnitude should be viewed as a rough approximation. Du et al. [2], in their meta-analysis of 11 studies involving 4,039 patients, reported an overall kinesiophobia prevalence of 35% after TKA, with pain, low social support, and negative coping strategies identified as key influencing factors. These findings underscore the multifactorial nature of kinesiophobia and suggest that exercise interventions may need to be complemented by strategies addressing these psychosocial determinants. Furthermore, the cross-sectional network analysis by Zhou et al. [34] identified three central components of kinesiophobia (items related to movement avoidance, perceived bodily risk, and somatic alarm) as well as rumination and coping self-efficacy as bridge variables connecting pain catastrophizing and kinesiophobia, providing potential targets for precision intervention. Compared with previous systematic reviews that have predominantly focused on multidisciplinary care incorporating psychological counseling, pharmacological management, and physical therapy [35], our study specifically isolates the effects of exercise-based interventions, thereby providing clearer evidence regarding the standalone efficacy of structured physical activity protocols in reducing kinesiophobia. Future research should prioritize dose-response designs to establish the minimum effective dose required to achieve the minimal clinically important difference for kinesiophobia.

A noteworthy finding from the modality-based subgroup analysis is that interventions incorporating cognitive restructuring or psychological techniques did not confer additional benefits over conventional exercise alone for reducing kinesiophobia. This finding does not constitute evidence that cognitive augmentation is ineffective. Rather, the apparent lack of statistically superior effect most likely reflects both insufficient statistical power (the cognitive/psychological subgroup comprised fewer studies with smaller aggregate sample sizes) and marked heterogeneity in the nature and intensity of the cognitive components across studies (e.g., from brief relaxation to complex motor imagery). None of the included trials reported baseline pain catastrophizing scores or adherence to the psychological modules, preventing any analysis by baseline psychological risk profile. Nevertheless, we propose a plausible explanatory hypothesis for future testing: the active therapeutic ingredient may be exposure-based desensitization: that is, patients learn through actual movement that physical activity does not cause harm, rather than the cognitive reframing per se. This interpretation aligns with the fear-avoidance model, which posits that behavioral exposure is the critical mechanism for breaking the fear-avoidance cycle. If this hypothesis holds, it would suggest that adding resource-intensive psychological modules to exercise regimens may not be necessary for the general TKA population. However, baseline psychological risk stratification might still identify subsets of patients who could benefit, and future trials should stratify randomization by baseline kinesiophobia severity to test this hypothesis.

### Possible dissociation between function and pain: an interpretable mid-term functional signal versus a fragile early pain signal

We observed a possible, hypothesis-generating divergence between knee function and pain outcomes, with different degrees of certainty across follow-up windows. For knee function, only the 12-week subgroup estimate was interpretable and statistically significant. The ≤ 4-week and ≥ 24-week functional subgroup estimates were not interpretable because of extreme heterogeneity; therefore, short- and longer-term functional effects remain uncertain. For pain, the ≤ 4-week finding should be regarded as a possible but non-robust signal rather than an established early analgesic effect: this estimate was not interpretable due to extreme heterogeneity and became non-significant after exclusion of the acute in-hospital trial by Núñez-Cortés 2024 [30]. At ≥ 24 weeks, no significant between-group difference was observed. Thus, the current evidence does not support a definitive temporally confined analgesic effect; it only suggests a fragile early pain signal that requires further confirmation.

If the interpretable 12-week functional benefit is real, it may reflect cumulative neuromuscular adaptations, including quadriceps activation, proprioceptive improvement, and recovery of gait biomechanics. Wen et al. [15] reported that kinesiophobia predicted function early (1–3 months), whereas function predicted kinesiophobia later (3–6 months), supporting phased rehabilitation strategies. The absence of a significant long-term analgesic effect should not be overinterpreted as evidence that exercise cannot relieve pain; floor effects and treatment contamination at later follow-ups may explain this finding. Heegaard et al. [36] found no reproducible psychological predictor of long-term pain outcomes. Future trials should distinguish acute postoperative nociceptive pain (≤ 3 months) from chronic postsurgical pain (> 3 months) using standardized temporal criteria.

### Secondary outcomes: preliminary signals requiring confirmation

For balance (Berg Balance Scale), time to first mobilization, and rehabilitation adherence, our pooled analyses yielded directionally favorable results. However, it is imperative to highlight that each of these outcomes was derived from only two small-scale trials. Despite reaching statistical significance, these estimates are fragile and susceptible to type I error.

The qualitative meta-synthesis by Chen et al. [16], which included 19 studies involving 205 TKA patients, identified fear of movement as one of the six key barriers to rehabilitation exercise adherence, alongside physical symptoms, cognitive biases, lack of rehabilitation-related knowledge, psychological stress, and socio-environmental constraints. The primary value of these findings is not to inform clinical practice but to advocate for the inclusion of objectively monitored adherence (e.g., via wearable accelerometers) and standardized mobilization timelines as core secondary endpoints in future large-scale RCTs, rather than treating them as ancillary records.

### Methodological limitations and evidence uncertainty

The interpretation of our findings must be tempered by several methodological constraints that collectively downgraded the GRADE certainty to low. First, clinical heterogeneity was substantial: interventions varied in modality, intensity, and frequency without standardized protocols, precluding specific exercise prescriptions. Second, psychometric nonequivalence across TSK‑11, TSK‑13, and TSK‑17 may have introduced measurement‑related heterogeneity. Although SMD standardization enables pooling across instruments with different score ranges, it cannot resolve differences in item composition and underlying factor structure across TSK variants. Third, lack of participant and personnel blinding (only three studies reported assessor blinding) likely inflated effect sizes for subjective outcomes. Fourth, the DerSimonian‑Laird random‑effects estimator implemented in RevMan 5.4 may underestimate between‑study variance under conditions of extreme heterogeneity (*I*^*2*^ > 90%), producing overly narrow confidence intervals. Future meta-analytic updates on this topic should consider restricted maximum-likelihood (REML) models together with Hartung‑Knapp‑Sidik‑Jonkman (HKSJ) adjusted confidence intervals to better quantify statistical uncertainty [37, 38]. Fifth, one included study [17] reported TSK scores as median (IQR) for the primary outcome and therefore required Wan et al. imputation [22]. Although this method is well validated, imputation from non-normal summaries may have introduced additional measurement-related heterogeneity. Sixth, although the search was unrestricted by language, our inclusion criteria restricted studies to English or Chinese publications, which may have excluded relevant studies published in other languages and introduced potential publication bias.

### Implications for clinical practice and future research

Based on this low-certainty evidence, we offer conservative yet actionable guidance for both clinical practice and future investigative efforts. In clinical settings, exercise-based rehabilitation may be considered as a low-risk adjunct to routine postoperative rehabilitation for patients with elevated baseline kinesiophobia, particularly during the early postoperative period. However, clinicians must communicate transparently that the evidence base is limited and uncertain. The current data suggest a possible short-term benefit on kinesiophobia and an interpretable 12-week functional signal, whereas short- and longer-term functional benefits remain uncertain. The true effects, if any, are likely smaller than the meta-analytic point estimates suggest, and individual responses are unpredictable. Given the null finding in the overall population for cognitive augmentation (likely due to underpowering), we caution against the indiscriminate application of resource-intensive psychological modules to all TKA patients. Instead, such modules should be reserved for patients with confirmed high baseline catastrophizing or kinesiophobia, pending better evidence. For patients with elevated baseline kinesiophobia, clinicians may consider graded resistance and graded‑exposure elements, particularly during the early postoperative period. However, the low certainty of the supporting evidence precludes any fixed protocol, and individualization based on clinical judgement and patient response remains essential. Regarding research directions, dose-response RCTs are urgently needed to define the minimum effective dose, and subsequent trials must adhere strictly to CONSORT guidelines with emphasis on allocation concealment and assessor blinding. Mixed-methods designs incorporating qualitative interviews may elucidate patient-level barriers to adherence, and we advocate for routine use of wearable technology to objectively monitor exercise adherence, replacing self-reported indicators. Finally, pre-registered subgroup analyses based on baseline catastrophizing and kinesiophobia severity are warranted to identify which patients benefit most from specific modalities. Collectively, these efforts would facilitate the translation of exercise prescriptions from a one-size-fits-all approach to a precision-based rehabilitation strategy.

## Conclusion

In summary, low-certainty evidence suggests that exercise-based rehabilitation may be associated with reductions in kinesiophobia at ≤ 4 weeks and an interpretable 12-week improvement in knee function after TKA; the ≤ 4-week and ≥ 24-week functional estimates were not interpretable because of extreme heterogeneity, and the early pain finding was a possible but non-robust signal. The evidence is limited by high unexplained heterogeneity, risk of bias from lack of blinding, and imprecision, and pooled effect sizes are likely overestimates. Exercise-based rehabilitation should currently be viewed as a low-risk, individualized adjunct for selected patients with elevated baseline fear of movement, not as a universal standard. Definitive validation awaits well-designed, multicenter RCTs with standardized protocols, assessor blinding, objective adherence monitoring, and sufficient power.

## Supplementary Information

Below is the link to the electronic supplementary material.

Supplementary Material 1

Supplementary Material 2

Supplementary Material 3

Supplementary Material 4

## Data Availability

The datasets used and/or analysed during the current study are available from the corresponding author on reasonable request. All data extracted from included studies are presented in the main manuscript and its supplementary information files.

## Declarations

### Systematic review registration

PROSPERO CRD420261461385

## List of abbreviations

BBS: Berg Balance Scale
CBM: China Biomedical Literature Database
CI: confidence interval
CINAHL: Cumulative Index to Nursing and Allied Health Literature
CNKI: China National Knowledge Infrastructure
CONSORT: Consolidated Standards of Reporting Trials
FPS‑R: Faces Pain Scale‑Revised
GRADE: Grading of Recommendations Assessment, Development and Evaluation
HSS: Hospital for Special Surgery Knee Score
KOOS: Knee Injury and Osteoarthritis Outcome Score
KSS: Knee Society Score
MD: mean difference
MR: mixed reality
NPRS: Numerical Pain Rating Scale
NRS: Numerical Rating Scale
OR: odds ratio
PMR: progressive muscle relaxation
PRISMA: Preferred Reporting Items for Systematic Reviews and Meta‑Analyses
PROSPERO: International Prospective Register of Systematic Reviews
RCT: randomized controlled trial
SMD: standardized mean difference
TKA: total knee arthroplasty
TSK: Tampa Scale for Kinesiophobia
VAS: Visual Analog Scale
WOMAC: Western Ontario and McMaster Universities Osteoarthritis Index
