# Supplementary material for: Exercise-based rehabilitation interventions for kinesiophobia after total knee arthroplasty: a systematic review and meta-analysis of randomized controlled trials
Source: BMC Musculoskelet Disord. 2026 Oct 21;27:923. doi: 10.1186/s12891-026-10640-3 (PMC13718169; doi:10.1186/s12891-026-10640-3)
Supplement: Supplementary file 1 [file 12891_2026_10640_MOESM1_ESM.docx]

**Search strategy for ten databases**

1. PubMed: 39 Results

#1 Arthroplasty, Replacement, Knee[Mesh]

#2 Arthroplasties, Replacement, Knee[Title/Abstract] OR Arthroplasty, Knee Replacement[Title/Abstract] OR Knee Replacement Arthroplasties[Title/Abstract] OR Knee Replacement Arthroplasty[Title/Abstract] OR Replacement Arthroplasties, Knee[Title/Abstract] OR Replacement Arthroplasty, Knee[Title/Abstract] OR Replacement, Total Knee[Title/Abstract] OR Total Knee Replacement[Title/Abstract] OR Knee Replacement, Total[Title/Abstract] OR Knee Arthroplasty[Title/Abstract] OR Arthroplasty, Knee[Title/Abstract] OR Arthroplasties, Knee Replacement[Title/Abstract] OR Knee Arthroplasty, Total[Title/Abstract] OR Arthroplasty, Total Knee[Title/Abstract] OR Total Knee Arthroplasty[Title/Abstract] OR Arthroplasty, Replacement, Partial Knee[Title/Abstract] OR Unicompartmental Knee Arthroplasty[Title/Abstract] OR Arthroplasty, Unicompartmental Knee[Title/Abstract] OR Knee Arthroplasty, Unicompartmental[Title/Abstract] OR Unicompartmental Knee Replacement[Title/Abstract] OR Knee Replacement, Unicompartmental[Title/Abstract] OR Partial Knee Replacement[Title/Abstract] OR Knee Replacement, Partial[Title/Abstract] OR Unicondylar Knee Replacement[Title/Abstract] OR Knee Replacement, Unicondylar[Title/Abstract] OR Unicondylar Knee Arthroplasty[Title/Abstract] OR Arthroplasty, Unicondylar Knee[Title/Abstract] OR Knee Arthroplasty, Unicondylar[Title/Abstract] OR Partial Knee Arthroplasty[Title/Abstract] OR Arthroplasty, Partial Knee[Title/Abstract] OR Knee Prosthesis[Title/Abstract]

#3 #1 OR #2

#4 Exercise Therapy[Mesh] OR Exercise Movement Techniques[Mesh] OR Physical Therapy Modalities[Mesh] OR Rehabilitation[Mesh:NoExp]

#5 exercise*[tiab] OR kinesiotherap*[tiab] OR therapeutic exercise[tiab] OR physical therapy[tiab] OR physiotherapy*[tiab] OR rehabilitation exercise*[tiab] OR exercise training[tiab] OR strength training[tiab] OR resistance training[tiab] OR aerobic exercise*[tiab] OR balance training[tiab] OR proprioceptive training[tiab] OR land-based exercise*[tiab] OR water-based exercise*[tiab] OR aquatic exercise*[tiab] OR aquatic therapy[tiab] OR hydrotherapy[tiab] OR postoperative exercise*[tiab] OR functional training[tiab] OR home-based exercise*[tiab] OR supervised exercise*[tiab] OR neuromuscular training[tiab] OR gait training[tiab] OR movement therapy[tiab] OR Tai Chi[tiab] OR Taiji[tiab] OR yoga[tiab] OR Pilates[tiab] OR progressive muscle relaxation[tiab] OR virtual reality[tiab] OR virtual reality rehabilitation[tiab] OR telerehabilitation[tiab] OR remote rehabilitation[tiab]

#6 #4 OR #5

#7 kinesiophob*[tiab] OR kinesophob*[tiab] OR fear of movement[tiab] OR movement fear[tiab] OR fear of re-injury[tiab] OR fear of reinjury[tiab] OR tampa scale*[tiab] OR TSK[tiab] OR pain-related fear[tiab]

#8 #3 AND #6 AND #7

2. Embase: 103 Results

#1 'knee replacement'/exp

#2 'arthroplasty, replacement, knee':ti,ab,kw OR 'knee joint replacement':ti,ab,kw OR 'knee joint replacements':ti,ab,kw OR 'knee replacement arthroplasty':ti,ab,kw OR 'knee replacements':ti,ab,kw OR 'knee replacement':ti,ab,kw

#3 #1 OR #2

#4 'kinesiotherapy'/exp OR 'physiotherapy'/exp OR 'rehabilitation'/exp

#5 'exercise*':ti,ab,kw OR 'kinesiotherap*':ti,ab,kw OR 'therapeutic exercise':ti,ab,kw OR 'physical therapy':ti,ab,kw OR 'physiotherapy*':ti,ab,kw OR 'rehabilitation exercise*':ti,ab,kw OR 'exercise training':ti,ab,kw OR 'strength training':ti,ab,kw OR 'resistance training':ti,ab,kw OR 'aerobic exercise*':ti,ab,kw OR 'balance training':ti,ab,kw OR 'proprioceptive training':ti,ab,kw OR 'land-based exercise*':ti,ab,kw OR 'water-based exercise*':ti,ab,kw OR 'aquatic exercise*':ti,ab,kw OR 'aquatic therapy':ti,ab,kw OR 'hydrotherapy':ti,ab,kw OR 'postoperative exercise*':ti,ab,kw OR 'functional training':ti,ab,kw OR 'home-based exercise*':ti,ab,kw OR 'supervised exercise*':ti,ab,kw OR 'neuromuscular training':ti,ab,kw OR 'gait training':ti,ab,kw OR 'movement therapy':ti,ab,kw OR 'tai chi':ti,ab,kw OR 'taiji':ti,ab,kw OR 'yoga':ti,ab,kw OR 'pilates':ti,ab,kw OR 'progressive muscle relaxation':ti,ab,kw OR 'virtual reality':ti,ab,kw OR 'virtual reality rehabilitation':ti,ab,kw OR 'telerehabilitation':ti,ab,kw OR 'remote rehabilitation':ti,ab,kw

#6 #4 OR #5

#7 'kinesiophob*':ti,ab,kw OR 'kinesophob*':ti,ab,kw OR 'fear of movement':ti,ab,kw OR 'movement fear':ti,ab,kw OR 'fear of re-injury':ti,ab,kw OR 'fear of reinjury':ti,ab,kw OR 'tampa scale*':ti,ab,kw OR 'TSK':ti,ab,kw OR 'pain-related fear':ti,ab,kw

#8 #3 AND #6 AND #7

3. Web of Science: 105 Results

#1 Arthroplasties, Replacement, Knee OR Arthroplasty, Knee Replacement OR Knee Replacement Arthroplasties OR Knee Replacement Arthroplasty OR Replacement Arthroplasties, Knee OR Replacement Arthroplasty, Knee OR Replacement, Total Knee OR Total Knee Replacement OR Knee Replacement, Total OR Knee Arthroplasty OR Arthroplasty, Knee OR Arthroplasties, Knee Replacement OR Knee Arthroplasty, Total OR Arthroplasty, Total Knee OR Total Knee Arthroplasty OR Arthroplasty, Replacement, Partial Knee OR Unicompartmental Knee Arthroplasty OR Arthroplasty, Unicompartmental Knee OR Knee Arthroplasty, Unicompartmental OR Unicompartmental Knee Replacement OR Knee Replacement, Unicompartmental OR Partial Knee Replacement OR Knee Replacement, Partial OR Unicondylar Knee Replacement OR Knee Replacement, Unicondylar OR Unicondylar Knee Arthroplasty OR Arthroplasty, Unicondylar Knee OR Knee Arthroplasty, Unicondylar OR Partial Knee Arthroplasty OR Arthroplasty, Partial Knee OR Knee Prosthesis (Topic)

#2 Exercise Therapy OR Exercise Movement Techniques OR Physical Therapy Modalities OR Rehabilitation OR exercise* OR kinesiotherap* OR therapeutic exercise OR physical therapy OR physiotherapy* OR rehabilitation exercise* OR exercise training OR strength training OR resistance training OR aerobic exercise* OR balance training OR proprioceptive training OR land-based exercise* OR water-based exercise* OR aquatic exercise* OR aquatic therapy OR hydrotherapy OR postoperative exercise* OR functional training OR home-based exercise* OR supervised exercise* OR neuromuscular training OR gait training OR movement therapy OR Tai Chi OR Taiji OR yoga OR Pilates OR progressive muscle relaxation OR virtual reality OR virtual reality rehabilitation OR telerehabilitation OR remote rehabilitation (Topic)

#3 kinesiophob* OR kinesophob* OR fear of movement OR movement fear OR fear of re-injury OR fear of reinjury OR tampa scale* OR TSK OR pain-related fear (Topic)

#4 #1 AND #2 AND #3

4. Cochrane Library: 55 Results

#1 MeSH descriptor: [Arthroplasty, Replacement, Knee] explode all trees

#2 (Knee Replacement Arthroplasties):ti,ab,kw OR (Arthroplasty, Knee):ti,ab,kw OR (Arthroplasty, Total Knee):ti,ab,kw OR (Arthroplasties, Knee Replacement):ti,ab,kw OR (Replacement Arthroplasties, Knee):ti,ab,kw OR (Total Knee Replacement):ti,ab,kw OR (Replacement, Total Knee):ti,ab,kw OR (Knee Arthroplasty):ti,ab,kw OR (Knee Replacement, Total):ti,ab,kw OR (Total Knee Arthroplasty):ti,ab,kw OR (Arthroplasty, Knee Replacement):ti,ab,kw OR (Knee Arthroplasty, Total):ti,ab,kw OR (Arthroplasties, Replacement, Knee):ti,ab,kw OR (Knee Replacement Arthroplasty):ti,ab,kw OR (Replacement Arthroplasty, Knee):ti,ab,kw OR (Arthroplasty, Unicondylar Knee):ti,ab,kw OR (Knee Replacement, Partial):ti,ab,kw OR (Unicompartmental Knee Arthroplasty):ti,ab,kw OR (Arthroplasty, Unicompartmental Knee):ti,ab,kw OR (Unicondylar Knee Arthroplasty):ti,ab,kw OR (Knee Arthroplasty, Unicondylar):ti,ab,kw OR (Partial Knee Arthroplasty):ti,ab,kw OR (Knee Arthroplasty, Partial):ti,ab,kw OR (Arthroplasty, Partial Knee):ti,ab,kw OR (Knee Replacement, Unicompartmental):ti,ab,kw OR (Unicompartmental Knee Replacement):ti,ab,kw OR (Knee Replacement, Unicondylar):ti,ab,kw OR (Knee Arthroplasty, Unicompartmental):ti,ab,kw OR (Arthroplasty, Replacement, Partial Knee):ti,ab,kw OR (Partial Knee Replacement):ti,ab,kw OR (Unicondylar Knee Replacement):ti,ab,kw

#3 #1 OR #2

#4 [Exercise Therapy] explode all trees OR [Exercise Movement Techniques] explode all trees OR [Physical Therapy Modalities] explode all trees OR [Rehabilitation] explode all trees

#5 (exercise*):ti,ab,kw OR (kinesiotherap*):ti,ab,kw OR (therapeutic exercise):ti,ab,kw OR (physical therapy):ti,ab,kw OR (physiotherapy*):ti,ab,kw OR (rehabilitation exercise*):ti,ab,kw OR (exercise training):ti,ab,kw OR (strength training):ti,ab,kw OR (resistance training):ti,ab,kw OR (aerobic exercise*):ti,ab,kw OR (balance training):ti,ab,kw OR (proprioceptive training):ti,ab,kw OR (land-based exercise*):ti,ab,kw OR (water-based exercise*):ti,ab,kw OR (aquatic exercise*):ti,ab,kw OR (aquatic therapy):ti,ab,kw OR (hydrotherapy):ti,ab,kw OR (postoperative exercise*):ti,ab,kw OR (functional training):ti,ab,kw OR (home-based exercise*):ti,ab,kw OR (supervised exercise*):ti,ab,kw OR (neuromuscular training):ti,ab,kw OR (gait training):ti,ab,kw OR (movement therapy):ti,ab,kw OR (Tai Chi):ti,ab,kw OR (Taiji):ti,ab,kw OR (yoga):ti,ab,kw OR (Pilates):ti,ab,kw OR (progressive muscle relaxation):ti,ab,kw OR (virtual reality):ti,ab,kw OR (virtual reality rehabilitation):ti,ab,kw OR (telerehabilitation):ti,ab,kw OR (remote rehabilitation):ti,ab,kw

#6 #4 OR #5

#7 MeSH descriptor: [Kinesiophobia] explode all trees

#8 (kinesiophob*):ti,ab,kw OR (kinesophob*):ti,ab,kw OR (fear of movement):ti,ab,kw OR (movement fear):ti,ab,kw OR (fear of re-injury):ti,ab,kw OR (fear of reinjury):ti,ab,kw OR (tampa scale*):ti,ab,kw OR (TSK):ti,ab,kw OR (pain-related fear):ti,ab,kw

#9 #7 OR #8

#10 #3 AND #6 AND #9

5. OVID MEDLINE: 28 Results

| 1 | Arthroplasty, Replacement, Knee/ |
| --- | --- |

2 (arthroplasty, replacement, knee or arthroplasties, replacement, knee or arthroplasty, knee replacement or knee replacement arthroplasties or knee replacement arthroplasties, knee or replacement arthroplasty, knee or replacement, total knee or total knee replacement or knee replacement, total or knee arthroplasty or arthroplasty, knee or arthroplasties, knee replacement or knee arthroplasty, total or arthroplasty, total knee or total knee arthroplasty or arthroplasty, replacement, partial knee or unicompartmental knee arthroplasty or arthroplasty, unicompartmental knee or knee arthroplasty, unicompartmental or unicompartmental knee replacement or knee replacement, unicompartmental or partial knee replacement or knee replacement, partial or unicondylar knee replacement or knee replacement, unicondylar or unicondylar knee arthroplasty or arthroplasty, unicondylar knee or knee arthroplasty, unicondylar or partial knee arthroplasty or arthroplasty, partial knee or knee arthroplasty, partial).ab,ti,kw.

3 1 or 2

4 Exercise Therapy/ OR Exercise Movement Techniques/ OR Physical Therapy Modalities/ OR Rehabilitation/

5 (exercise* OR kinesiotherap* OR therapeutic exercise OR physical therapy OR physiotherapy* OR rehabilitation exercise* OR exercise training OR strength training OR resistance training OR aerobic exercise* OR balance training OR proprioceptive training OR land-based exercise* OR water-based exercise* OR aquatic exercise* OR aquatic therapy OR hydrotherapy OR postoperative exercise* OR functional training OR home-based exercise* OR supervised exercise* OR neuromuscular training OR gait training OR movement therapy OR Tai Chi OR Taiji OR yoga OR Pilates OR progressive muscle relaxation OR virtual reality OR virtual reality rehabilitation OR telerehabilitation OR remote rehabilitation).ab,ti,kw.

6 4 or 5

7 (kinesiophob* OR kinesophob* OR fear of movement OR movement fear OR fear of re-injury OR fear of reinjury OR tampa scale* OR TSK OR pain-related fear).ab,ti,kw.

8 3 or 6 or 7

6. CINAHL: 10 Results

S1 SU (Arthroplasties, Replacement, Knee OR Arthroplasty, Knee Replacement OR Knee Replacement Arthroplasties OR Knee Replacement Arthroplasty OR Replacement Arthroplasties, Knee OR Replacement Arthroplasty, Knee OR Replacement, Total Knee OR Total Knee Replacement OR Knee Replacement, Total OR Knee Arthroplasty OR Arthroplasty, Knee OR Arthroplasties, Knee Replacement OR Knee Arthroplasty, Total OR Arthroplasty, Total Knee OR Total Knee Arthroplasty OR Arthroplasty, Replacement, Partial Knee OR Unicompartmental Knee Arthroplasty OR Arthroplasty, Unicompartmental Knee OR Knee Arthroplasty, Unicompartmental OR Unicompartmental Knee Replacement OR Knee Replacement, Unicompartmental OR Partial Knee Replacement OR Knee Replacement, Partial OR Unicondylar Knee Replacement OR Knee Replacement, Unicondylar OR Unicondylar Knee Arthroplasty OR Arthroplasty, Unicondylar Knee OR Knee Arthroplasty, Unicondylar OR Partial Knee Arthroplasty OR Arthroplasty, Partial Knee OR Knee Prosthesis)

S2 SU (Exercise Therapy OR Exercise Movement Techniques OR Physical Therapy Modalities OR Rehabilitation OR exercise* OR kinesiotherap* OR therapeutic exercise OR physical therapy OR physiotherapy* OR rehabilitation exercise* OR exercise training OR strength training OR resistance training OR aerobic exercise* OR balance training OR proprioceptive training OR land-based exercise* OR water-based exercise* OR aquatic exercise* OR aquatic therapy OR hydrotherapy OR postoperative exercise* OR functional training OR home-based exercise* OR supervised exercise* OR neuromuscular training OR gait training OR movement therapy OR Tai Chi OR Taiji OR yoga OR Pilates OR progressive muscle relaxation OR virtual reality OR virtual reality rehabilitation OR telerehabilitation OR remote rehabilitation)

S3 SU (kinesiophob* OR kinesophob* OR fear of movement OR movement fear OR fear of re-injury OR fear of reinjury OR tampa scale* OR TSK OR pain-related fear)

S4 S1 AND S2 AND S3

7. China National Knowledge Infrastructure: 44 Results

SU=('全膝关节置换' + '全膝关节置换术' + '全膝置换' + '全膝置换术' + '人工全膝关节置换' + '人工全膝关节置换术' + 'TKA' + 'TKR' + '全膝关节表面置换' + '膝关节表面置换术' + '人工膝关节置换' + '人工膝关节置换术') AND SU=('运动疗法' + '运动干预' + '运动训练' + '康复运动' + '功能锻炼' + '物理治疗' + '康复训练 +渐进性运动' + '抗阻运动' + '有氧运动' + '平衡训练' + '水中运动' + '太极拳' + '瑜伽' + '虚拟现实康复' + '远程康复' + '普拉提') AND SU=('恐动症' + '运动恐惧' + '运动恐惧症' + '活动恐惧' + '恐惧回避' + 'TSK' + '坦帕运动恐惧量表' + '坦帕恐动量表')

8. Wanfang 98 Results

主题:(全膝关节置换 OR 全膝关节置换术 OR 全膝置换 OR 全膝置换术 OR 人工全膝关节置换 OR 人工全膝关节置换术 OR TKA OR TKR OR 全膝关节表面置换 OR 膝关节表面置换术 OR 人工膝关节置换 OR 人工膝关节置换术) and 主题:(运动疗法 OR 运动干预 OR 运动训练 OR 康复运动 OR 功能锻炼 OR 物理治疗 OR 康复训练 OR渐进性运动 OR 抗阻运动 OR 有氧运动 OR 平衡训练 OR 水中运动 OR 太极拳 OR 瑜伽 OR 虚拟现实康复 OR 远程康复 OR 普拉提) and 主题:(恐动症 OR 运动恐惧 OR 运动恐惧症 OR 活动恐惧 OR 恐惧回避 OR TSK OR 坦帕运动恐惧量表 OR 坦帕恐动量表)

9. VIP: 18 Results

M=(全膝关节置换 OR 全膝关节置换术 OR 全膝置换 OR TKA OR TKR OR 人工膝关节置换) and M=(可穿戴设备 OR 可穿戴传感器 OR 智能手环 OR 智能手表 OR 加速度计 OR 惯性传感器 OR IMU OR 计步器 OR 活动追踪器 OR 移动医疗 OR mHealth)

10. China Biomedical Literature Database: 59 Results

( "全膝关节置换"[常用字段:智能] OR "全膝关节置换术"[常用字段:智能] OR "全膝置换"[常用字段:智能] OR "全膝置换术"[常用字段:智能] OR "人工全膝关节置换"[常用字段:智能] OR "人工全膝关节置换术"[常用字段:智能] OR "TKA"[常用字段:智能] OR "TKR"[常用字段:智能] OR "全膝关节表面置换"[常用字段:智能] OR "膝关节表面置换术"[常用字段:智能] OR "人工膝关节置换"[常用字段:智能] OR "人工膝关节置换术"[常用字段:智能]) AND( "运动疗法"[常用字段:智能] OR "运动干预"[常用字段:智能] OR "运动训练"[常用字段:智能] OR "康复运动"[常用字段:智能] OR "功能锻炼"[常用字段:智能] OR "物理治疗"[常用字段:智能] OR "康复训练 OR渐进性运动"[常用字段:智能] OR "抗阻运动"[常用字段:智能] OR "有氧运动"[常用字段:智能] OR "平衡训练"[常用字段:智能] OR "水中运动"[常用字段:智能] OR "太极拳"[常用字段:智能] OR "瑜伽"[常用字段:智能] OR "虚拟现实康复"[常用字段:智能] OR "远程康复"[常用字段:智能] OR "普拉提"[常用字段:智能]) AND( "恐动症"[常用字段:智能] OR "运动恐惧"[常用字段:智能] OR "运动恐惧症"[常用字段:智能] OR "活动恐惧"[常用字段:智能] OR "恐惧回避"[常用字段:智能] OR "TSK"[常用字段:智能] OR "坦帕运动恐惧量表"[常用字段:智能] OR "坦帕恐动量表"[常用字段:智能])
