# Supplementary material for: Exercise-based rehabilitation interventions for kinesiophobia after total knee arthroplasty: a systematic review and meta-analysis of randomized controlled trials
Source: BMC Musculoskelet Disord. 2026 Oct 21;27:923. doi: 10.1186/s12891-026-10640-3 (PMC13718169; doi:10.1186/s12891-026-10640-3)
Supplement: Supplementary file 2 [file 12891_2026_10640_MOESM2_ESM.docx]

**Full-text articles excluded and reasons for exclusion (n=19)**

| No. | Title | Reason for exclusion |
| --- | --- | --- |
| 1 | The Effect of Mobile-based Education and Exercise Program Given to Patients With Total Knee Replacement Surgery on Anxiety, Kinesiophobia, and Physical Function | Insufficient extractable outcome data |
| 2 | The Effect of Graded Motor Imagery Training on Pain, Functional Performance, Motor Imagery Skill, and Kinesiophobia After Total Knee Arthroplasty | Insufficient extractable outcome data |
| 3 | The Effects of Elastic Band Exercise on the Pain, Kinesiophobia, Functional, and Psychological Status After Total Knee Arthroplasty | Insufficient extractable outcome data |
| 4 | Efficacy of Proprioceptive Neuromuscular Facilitation Exercise Programme Versus Usual Care in Patients With Total Knee Arthroplasty: A Randomised Controlled Trial | Insufficient extractable outcome data |
| 5 | The Effect of Virtual Reality on Pain, Kinesiophobia and Function in Total Knee Arthroplasty Patients | Insufficient extractable outcome data |
| 6 | Effects of Motor Imagery and Action Observation Training on Pain, Functionality, Balance and Fear of Movement in Total Knee Arthroplasty: A Randomized Controlled Trial | Insufficient extractable outcome data |
| 7 | Efficacy of Multifaceted Exercise Framework versus standard physiotherapy in Post Total Knee Arthroplasty patients | Insufficient extractable outcome data |
| 8 | Motor Imagery and Action Observation Training in Total Knee Arthroplasty: a Randomized Controlled Trial | Insufficient extractable outcome data |
| 9 | Effects of Progressive Restricted Blood Flow Exercises in Post Total Knee Arthroplasty | Insufficient extractable outcome data |
| 10 | Effect of Virtual Reality-Based Relaxation Exercise on Postoperative Pain and Kinesiophobia After Total Knee Replacement: Randomized Controlled Study | Ineligible control |
| 11 | The effect of graded motor imagery training on pain, functional performance, motor imagery skills, and kinesiophobia after total knee arthroplasty: randomized controlled trial | Ineligible control |
| 12 | Effects of Virtual Reality Technology on Analgesia for Rehabilitation Exercises and Rehabilitation Outcomes in Patients after Total Knee Arthroplasty | Ineligible control |
| 13 | The effect of virtual reality on pain, kinesiophobia and function in total knee arthroplasty patients: A randomized controlled trial | Ineligible control |
| 14 | The effect of adding motor imagery to acute rehabilitation program on pain, functional performance, motor imagery ability and kinesiophobia in patients who had received total knee arthroplasty | Ineligible control |
| 15 | Motor imagery enhances early rehabilitation in patients with high kinesiophobia after total knee arthroplasty: a randomized controlled trial | Ineligible control |
| 16 | Effect of Virtual Reality-Based Relaxation Exercise on Postoperative Pain and Kinesiophobia After Total Knee Replacement | Ineligible control |
| 17 | Does eccentric-concentric resistance training improve early functional outcomes compared to concentric resistance training after total knee arthroplasty? | Ineligible control |
| 18 | A Physical Therapist Administered Physical Activity Intervention After Total Knee Replacement | Inconsistent outcomes |
| 19 | Relaxation exercise therapy improves pain, muscle strength, and kinesiophobia following total knee arthroplasty in the short term: a randomized controlled trial | duplicate |
