# Supplementary material for: Exercise-based rehabilitation interventions for kinesiophobia after total knee arthroplasty: a systematic review and meta-analysis of randomized controlled trials
Source: BMC Musculoskelet Disord. 2026 Oct 21;27:923. doi: 10.1186/s12891-026-10640-3 (PMC13718169; doi:10.1186/s12891-026-10640-3)
Supplement: Supplementary file 3 [file 12891_2026_10640_MOESM3_ESM.docx]

**Subgroup estimates with extreme heterogeneity (*I^2^* > 90%).**

| Outcome | Subgroup | k | n | SMD (95% CI) | *I^2^* |
| --- | --- | --- | --- | --- | --- |
| Kinesiophobia | ≥ 24 weeks | 4 | 405 | −1.27 (−2.30 to −0.24) | 95% |
| Kinesiophobia | Digital/technology-assisted exercise | 2 | 190 | −1.12 (−2.23 to 0.00) | 92% |
| Knee function | ≤ 4 weeks | 3 | 208 | 2.05 (−0.02 to 4.12) | 97% |
| Knee function | ≥ 24 weeks | 3 | 307 | 1.49 (0.26 to 2.71) | 95% |
| Knee function | Conventional exercise | 4 | 445 | 2.28 (0.91 to 3.66) | 97% |
| Pain | ≤ 4 weeks | 4 | 408 | −1.09 (−2.03 to −0.14) | 94% |
| Pain | Conventional exercise | 2 | 260 | −1.22 (−3.63 to 1.19) | 98% |

k, number of studies; n, sample size; SMD, standardized mean difference; CI, confidence interval. All subgroups had *I²* > 90% and were considered not interpretable. Estimates are shown for transparency only and should not be interpreted as reliable significance-tested findings.
