# Supplementary material for: Exercise-based rehabilitation interventions for kinesiophobia after total knee arthroplasty: a systematic review and meta-analysis of randomized controlled trials
Source: BMC Musculoskelet Disord. 2026 Oct 21;27:923. doi: 10.1186/s12891-026-10640-3 (PMC13718169; doi:10.1186/s12891-026-10640-3)
Supplement: Supplementary file 4 [file 12891_2026_10640_MOESM4_ESM.docx]

**Summary of GRADE evidence quality and effect estimates**

| **Certainty assessment** | | | | | | | **№ of patients** | | **Effect** | | **Certainty** | **Importance** |
| --- | --- | --- | --- | --- | --- | --- | --- | --- | --- | --- | --- | --- |
| **№ of studies** | **Study design** | **Risk of bias** | **Inconsistency** | **Indirectness** | **Imprecision** | **Other considerations** | **Exercise-based rehabilitation** | **Routine rehabilitation** | **Relative (95% CI)** | **Absolute (95% CI)** |  |  |
| **Kinesiophobia** | | | | | | | | | | | | |
| 11 | randomised trials | serious^a^ | serious^b^ | not serious | not serious | none | 550 | 546 | - | SMD **1.22 lower** (1.72 lower to 0.72 lower) | ⨁⨁◯◯ Low^a,b^ | CRITICAL |
| **Knee joint function** | | | | | | | | | | | | |
| 10 | randomised trials | serious^a^ | serious^b^ | not serious | not serious | none | 501 | 497 | - | SMD **1.19 higher** (0.67 higher to 1.71 higher) | ⨁⨁◯◯ Low^a,b^ | CRITICAL |
| **pain** | | | | | | | | | | | | |
| 7 | randomised trials | serious^a^ | serious^b^ | not serious | not serious | none | 367 | 364 | - | SMD **0.71 lower** (1.21 lower to 0.21 lower) | ⨁⨁◯◯ Low^a,b^ | CRITICAL |
| **BBS** | | | | | | | | | | | | |
| 2 | randomised trials | serious^a^ | not serious | not serious | serious^c^ | none | 148 | 147 | - | MD **4.5 higher** (2.97 higher to 6.02 higher) | ⨁◯◯◯ Very Low^a,c^ | IMPORTANT |
| **Time to first mobilization** | | | | | | | | | | | | |
| 2 | randomised trials | serious^a^ | not serious | not serious | serious^c^ | none | 103 | 102 | - | MD **6.49 lower** (8.3 lower to 4.67 lower) | ⨁◯◯◯ Very Low^a,c^ | IMPORTANT |
| **Rehabilitation adherence** | | | | | | | | | | | | |
| 2 | randomised trials | serious^a^ | not serious | not serious | serious^c^ | none | 139/149（93.3%） | 110/149（73.8%） | **OR 5.11** (2.42 to 10.78) | **195 more per 1,000** (from 109 more to 266 more) | ⨁◯◯◯ Very Low^a,c^ | IMPORTANT |

**CI:** confidence interval; **MD:** mean difference; **OR:** odds ratio; **SMD:** standardised mean difference; **BBS:** Berg Balance Scale; **TSK:** Tampa Scale for Kinesiophobia

#### Explanations

a. **Risk of bias:** Performance bias was unavoidable due to the nature of exercise interventions (no participant/personnel blinding); allocation concealment was unclear in approximately half of included studies. Downgraded one level for serious risk of bias.

b. **Inconsistency:** Substantial between-study heterogeneity (I² ≥ 50%) was observed, driven by variability in intervention protocols, follow-up duration and outcome measurement instruments. Downgraded one level for serious inconsistency.

c. **Imprecision:** Each of these outcomes was based on only two small studies with limited total sample size and relatively wide confidence intervals. Downgraded two levels for very serious imprecision.
